# The Institutional Library

**Published:** 1920-08-21

**Authors:** 


### THE INSTITUTIONAL LIBRARY.

**The After-Treatment of Wounds and Injuries.** By R. C. Elmslie, M.S., F.R.C.S.Eng. With 144 illustrations. (London: J. and A. Churchill, 1919. Pp. vi.+323, 8vo. Price 15s. net.)

Mr. Elmslie has written in good English an excellent account of the experience he has acquired during the war in a military orthopædic hospital. Equipped at the commencement with special knowledge of orthopædic surgery, a trained anatomist and a competent pathologist, he was able to take a very wide view of the after-treatment of wounds and injuries. He has digested his experience in nineteen well-considered chapters, which range from the treatment of sinuses to physio-therapy and the technique of applying a plaster-of-Paris bandage. The book is made readable by the narration of cases which have come under the observation of the author, who describes the treatment adopted and the results attained. In all cases he adopts methods which appeal to common-sense, and rides no hobby. His remarks in connection with the difficult subject of functional disabilities are especially valuable, for he shows that they are not due to malingering, and he explains the manner in which these two conditions can be differentiated. He bears no malice towards bone-setters, but shows that better results are obtained by using their methods with knowledge than they themselves gain in their ignorance. The drawings are good, and it would have been well if Mr. Elmslie had stated in his preface the names of the artists employed to make them.

**The Diseases of Infancy and Childhood.** By L. Emmett Holt, M.D., and John Howland, M.D. (New York and London: D. Appleton and Co. 1919. Pp. 1,180. Price 35s. net.)

The seventh edition of this well-known text-book from across the Atlantic has undergone thorough revision and expansion to keep it up to date. As before, the key-note is thoroughness and common-sense. The authors are not afraid to express definite opinions on controversial topics; and the original author, Dr. Holt, has lived to see many of his views, unpopular when originally voiced, obtain general acceptance with the bulk of the British and American medical profession. The sections of a book on pediatrics by which the characteristics of the rest of the book can often be gauged are those on infant feeding. The authors rightly give this important topic a prominent position and a great deal of space. Their views are thoroughly sound; it may be noted in passing that they are not yet convinced by the advocates of four-hourly feeding for young infants, that they disapprove both of lime-water and citrate of soda as ingredients of bottle-feeds, and that their table of proprietary infant foods (with analyses) includes some which are unknown here and leaves out others which are very fashionable. As a thoroughly judicious and at the same time learned and comprehensive treatise on the diseases of childhood, this volume is hard to beat.

**Electric Ionisation.** A Practical Introduction to its Use in Medicine and Surgery. By A. R. Friel, M.A., M.D., F.R.C.S.I. (Bristol: John Wright and Sons, Ltd. London: Simpkin, Marshall, Hamilton, Kent and Co., Ltd. 1920. Cloth, 8s. net.)

A simple and readable little book which will provide valuable assistance to those who would attempt the use of this electrical method. Following a brief description of the physical principles involved, a reasoned summary is given of what can be expected from ionisation and the best methods by which it can be applied to the average cases met in general practice. As an introduction, this volume is strongly to be recommended.

**A British Nurse in Bolshevik Russia.** The Narrative of Margaret H. Barber. April 1916—December 1919. (London: A. C. Fifield. Price 1s. 6d. With a Foreword from the Rev. Harold Buxton.)

Miss Barber's narrative is exceedingly direct, simple, and unornamented. She left London for Russia in April 1916 with the Lord Mayor's Armenian Relief Pioneer Unit, and went from Petrograd to Van in Armenia till August, when the party disbanded on the approach of the Turks. She then joined the "Friends' War Victims' Committee" Unit, and worked in a village in the Samara Government for two years, right through the revolution and under Bolshevik rule. When the Unit broke up she went in June 1918 to Moscow, where she joined the Armenian Unit for refugees from the Caucasus, and twice journeyed to Petrograd for visits. Later she went to Astrakan with stores for a Bolshevik Armenian Hospital for refugees, where she stayed over a year. In October 1919 she made part of a Red Cross deputation for negotiating the repatriation of Armenian refugees, and was subsequently and with reluctance repatriated herself. The interest of the little book lies not so much in its matter as in its manner. It is markedly restrained and fair in tone. It is a plain tale of one who witnessed the life of the people in Russia at close quarters during the most lurid period and beheld no atrocities. But it is more than astonishing to read the calm narrative of this nurse who went on nursing whatever happened, and find her recording that no insult, outrage, or inhumanity occurred among the folk who came under her observation.

**Industrial Nursing.** By Florence Swift Wright, R.N. (The Macmillan Co., New York. 1919. Price 6s. 6d. net.)

America is still far ahead of this country in all matters of industrial welfare, and schemes there have approached well-nigh to perfection. The author of the above-mentioned volume is a woman of wide experience in all branches of welfare in her own country, and has put forward what is practically a handbook for this important branch of nursing. Every branch of factory nursing is discussed in general and in detail. Hints on the stocking and running of the ambulance room are much more helpful than the majority of such lists, and we are led on from these to the wider aspect of the nurse's duties and the inter-relationship which should exist between her and (1) her fellow-workers, (2) the community, and (3) her connection with the employment department of the factory. The factory nurse is urged, before taking up a position, to obtain some general knowledge of the conditions of industry and to secure some experience in the works employment department, so that she may gain an insight into the necessary procedures for selecting and engaging workers. Many will eventually widen their sphere of activities by actually taking control of the employment office, and, indeed, direct all industrial-welfare arrangements, but these will, of course, be the women who have taken pains to add to their qualifications and training. Hospital training alone is not enough.

